# Case report: Diagnosis and treatment of DGAT1 deficiency-induced congenital diarrhea in two cases and literature review

**DOI:** 10.3389/fped.2023.1253800

**Published:** 2023-10-16

**Authors:** Jian Li, Mei Sun, Jing Guo, Lingfen Xu

**Affiliations:** Department of Pediatrics, Shengjing Hospital of China Medical University, Shenyang, China

**Keywords:** diarrhea, congenital diarrhea, diacylglycerol acyltransferase 1 gene, growth retardation, hypoalbuminemia, DGAT1 deficiency

## Abstract

**Introduction:**

Congenital diarrhea is a rare inherited intestinal disease characterized by persistent and severe diarrhea and malabsorption in the first few weeks after birth, which can be life-threatening. Some congenital diarrheal diseases are associated with mutations in the diacylglycerol acyltransferase 1 *(DGAT1)* gene.

**Case descriptions:**

This study delineated 2 cases of diarrhea and growth retardation, subsequently confirmed as congenital diarrhea via genetic testing, revealing that the etiology involved compound heterozygous mutations in the DGAT1 gene.

**Diagnostic assessments:**

High-MCT milk powder did not obtain an ideal outcome, whereas low-fat diets improved the symptoms of diarrhea and increased the body weigths.

**Disscussion:**

The two cases facilitated our understanding of the clinical features of, and treatments for, patients harboring a DGAT1 mutation and enriched the existing DGAT1 mutation database.

## 1. Introduction

Congenital diarrhea is a single-gene hereditary disease characterized by severe diarrhea during the first few weeks after birth. According to the various pathological conditions, congenital diarrhea can be divided into five types (Table 1) (1) (1): diseases associated with epithelial nutrient/electrolyte transport disorders, (2) diseases related to epithelial cell enzymes and metabolic disorders, (3) diseases associated with transport and polarity disorders of epithelial cells, (4) diseases involving intestinal endocrine cell dysfunction, (5) enteropathy associated with immune disturbances. Children with severe congenital diarrheal diseases such as intestinal dysplasia/tufted enteropathy, microvilli inclusion disease, and Trichohepatoenteric syndrome, require prolonged parenteral nutrition or home parenteral nutrition (2, 3). Two cases of diacylglycerol acyltransferase 1 (DGAT1) deficiency have been described here. DGAT1 deficiency caused by a DGAT1 gene mutation can lead to aberrant lipid metabolism, resulting in congenital diarrhea and protein-losing enteropathy. To date, only a few cases of DGAT1 deficiency have been reported. These patients usually have severe diarrhea and symptom onset in the neonatal period or within 2–3 months of birth. Gene sequencing in the early stages of the disease can shorten the diagnostic time and allow children to be treated promptly.

**Table 1:** Congenital diarrhea and enteropathies.

Groups	Disease name	Gene name	Inheritance
Epithelial nutrient/electrolyte transport	Congenital chloride diarrhea	*SLC26A3*	AR
	Congenital sodium diarrhea	*SLC9A3*	AR
	Congenital sodium diarrhea	*GUCY2C*	AD
	Glucose-galactose malabsorption	*SLC5A1*	AR
	Primary bile acid diarrhea	*SLC10A2* *SLC51B*	AR
	Acrodermatitis enteropathica	*SLC39A4*	AR
Epithelial enzymes and metabolism	Congenital lactase deficiency	*LCT*	AR
	Sucrase-isomaltase deficiency	*SI*	AR
	Trehalase deficiency	*TREH*	AR
	Enterokinase deficiency	*TMPRSS15*	AR
	DGAT1 deficiency	*DGAT1*	AR
	PLVAP deficiency	*PLVAP*	AR
	Abetalipoproteinemia	*MTP*	AR
	Hypobetalipoproteinemia	*APOB*	AR
		*ANGPTL3*	AR
	Chylomicron retention disease	*SAR1B*	AR
	Dyskeratosis congenita	*TERT*	AR/AD
	Kabuki syndrome	*KMT2D*	AD
Epithelial trafficking and polarity	Microvillus inclusion disease	*MYO5B*	AR
		*STX3*	AR
	Tufting enteropathy	*EPCAM*	AR
	Syndromic Na + diarrhea	*SPINT2*	AR
	Trichohepatoenteric syndrome 1	*TTC37*	AR
	Trichohepatoenteric syndrome 2	*SKIV2l*	AR
	Familial hemophagocytic lymphohistiocytosis 5	*STXBP2*	AR
	TTC7A deficiency	*TTC7A*	AR
Enteroendocrine cell dysfunction	Enteric anendocrinosis	*NEUROG3*	AR
	X-linked lissencephaly and MR	*ARX*	X-linked
	Proprotein convertase 1/3 deficiency	*PCSK1*	AR
	Mitchell-Riley syndrome	*RFX6*	AR
Immune dysregulation-associated enteropathy	IPEX	*FOXP3*	X-linked
	ICOS deficiency	*ICOS*	AR
	ADAM17 deficiency	*ADAM17*	AR
	EGFR deficiency	*EGFR*	AR
	CD55 deficiency	*CD55*	AR
	CTLA4 deficiency	*CTLA4*	AD
	LRBA deficiency	*LRBA*	AR
	XIAP	*BIRC4*	X-linked

AD, autosomal dominant; AR, autosomal recessive.

## 2. Case descriptions

### 2.1. Case 1

A 3-month-old girl was brought to the Department of Pediatric Gastroenterology, Shengjing Hospital Affiliated with China Medical University, in May 2022 due to “no weight gain for 3 months, diarrhea for one day, and fever once”. She was primogenic to her mother with a full-term natural delivery, no history of asphyxia rescue, and normal gross motor development. Her birth weight was 2.75 kg, and she was fed standard formulas. At 2.5 months of age, the infant's weight was 2.5 kg. Her diet was changded to a high-calorie milk powder (whole protein formula powder, 100 kcal/100 ml) at 200 kcal/kg daily; however, her body weight continued to decrease. One day before admission, the infant had diarrhea, with large yellow loose stools four times a day with no mucusor blood. Additionally, she had a fever with minimal urine volume. She was admitted to the emergency department and hospitalized. After onset, the infant did not vomit with regular milk intake and sucked strongly. The infant could raise her head and exhibited normal gross motor development. Her parents were healthy and had no family history of genetic metabolic diseases.

Physical examination: body temperature: 39.1°C; pulse: 160 beats/min; breathing: 45 breaths/min; blood pressure: 84/43 mmHg; without oxygen uptake percutaneous oxygen saturation (SpO2) 98%; body weight: 2.2 kg (below the 3rd percentile); and body length 53 cm (below the 3rd percentile). The infant had a poor response status, manifesting as severe dehydration, sunken anterior fontanel, sunken eyes, pale and dull skin, thin subcutaneous fat, poor skin elasticity, soft abdomen without distension, no tenderness, normal bowel sounds, cool extremities, no oedema of both lower limbs, and no abnormalities in the cardiopulmonary and nervous systems during physical examination.

Auxiliary examination: laboratory tests revealed metabolic acidosis, serum ion disturbances, hypoproteinemia, hypogammaglobulinemia, and vitamin D deficiency (Table 2). Inflammatory markers, stool examination, abdominal CT, hepatobiliary and spleen ultrasound, urinary ultrasound, and cardiac ultrasound are normal.

**Table 2:** Laboratory test results of case 1 and case 2.

Laboratory tests	Case 1	Case 2
PH (7.35–7.45)	7.16	7.37
BE (−3-+3 mmol/L)	−13.4	−4.4
K^+^ (3.5–5.5 mmol/L)	3.2	3.0
Na^+^ (135–145 mmol/L)	126	133
Ca^+^ (1.9–2.6 mmol/L)	1.62	1.38
HGB (20–150 g/L)	137	63
TG (0.4–1.69 mmol/L)	1.5	3.72
Alb (35–53 g/L)	23.9	14.4
IgG (4.81–12.2 mmol/L	0.99	2.49
IgA (0.42–1.58 mmol/L)	0.123	0.185
IgM (0.41–1.65 mmol/L)	0.133	0.188
Total 25-hydroxyvitamin D (≥30 ng/ml)	7.43	3.23

BE, extracellular base excess; K^+^, potassium-ion; Na^+^, sodium-ion; Ca^+^, calcium-ion; HGB, hemoglobin; TG, triglycerides; Alb, albumin; IgG, immunoglobulin G; IgA, immunoglobulin A; IgM, immunoglobulin M.

### 2.2. Case 2

A 5-month-old boy was brought to the Department of Pediatric Gastroenterology, Shengjing Hospital Affiliated with China Medical University in November 2022 owing to “diarrhea and poor weight gain for 5 months and edema in both feet for 1 month”. The infant was an *in vitro* fertilization (IVF) baby delivered via full-term caesarean section with a birth weight of 3.7 kg. The patient had no history of asphyxia at birth. The infant was fed a mixed diet after birth and had diarrhea 7–8 times daily with yellow paste-like stool containing mucus and curds, vomiting, scattered rashes throughout the body, and insignificant weight gain. At the age of 2 months, the diarrhea was slightly relieved after switching to an amino acid formula, and the infant had loose stools containing mucus occurring 4–5 times a day. The rashes subsided. The infant was diagnosed with allergy to cow milk protein. Diarrhea, vomiting, and no weight gain were observed after three months. The infant developed pitting oedema in both feet at 4 months. He was in a poor mental state, crying easily, and had a low milk intake (100 kcal/kg every day). He suckled weakly, taking approximately 40 min per meal. The urine volume was normal.

Physical examination: body temperature: 36.8°C; pulse: 145 beats/min; respiration: 38 breaths/min; blood pressure: 87/46 mmHg; body weight: 4.0 kg (below the 3rd percentile); body length: 57 cm (below the 3rd percentile); poor response state, pale complexion, moderate dehydration appearance, sunken anterior fontanelle, poor skin elasticity, thin subcutaneous fat, slight oedema of eyelids, soft abdomen without distension, no tenderness, liver and spleen not palpable under the ribs, non-pitting oedema of both feet, and no abnormalities in the cardiopulmonary and nervous system examinations.

Auxiliary examination: Laboratory tests revealed serum ion disturbances, hypoproteinemia, hypogammaglobulinemia, increased triglyceride levels, and vitamin D deficiency (Table 2). Inflammatory markers, stool examination, abdominal CT, hepatobiliary and spleen ultrasound, urinary ultrasound, and cardiac ultrasound findings were normal.

## 3. Diagnostic assessment

### 3.1. Case 1

Diagnosis and treatment: After admission, the infant was initially diagnosed with severe acute diarrhea and malnutrition. The patient was treated with oral intestinal mucosa-protecting drugs (montmorillonite powder) to correct the dehydration and ion disorder. Lactose-free regular formula milk powder was administered via nasal feeding and continuously pumped for 24 h, but the symptoms did not decrease. Subsequently, IV nutritional support was administered. The patient was gradually transitioned to a formula powder containing 50% medium-chain triglyceride (MCT) and 48% of the total energy provided by fat after the alleviation of the diarrhea with yellow paste-like stool 2–3 times daily. The baby's family declined a colonoscopy and pathological examination during hospitalization. On the 19th day of admission, the diarrhea symptoms improved and she was discharged with a body weight of 2.8 kg.

**Figure 1:**
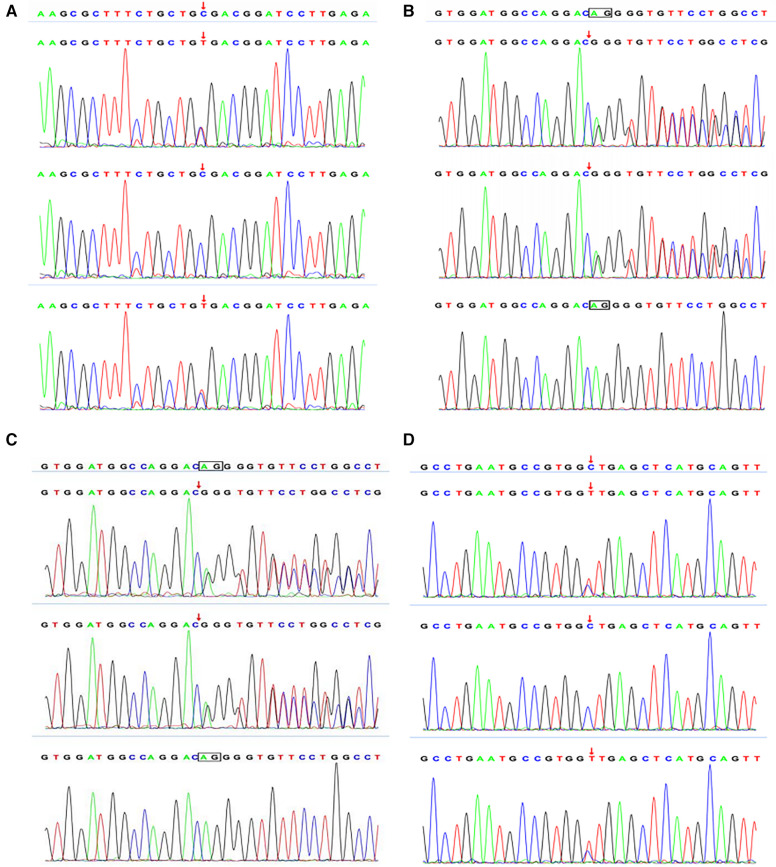
Singer verification of case 1: the child shown in (**A**) has a mutation in the c.1215_1216delAG site of DGAT1 gene, and the mutation comes from his mother. The child shown in (**B**) has a mutation in the c.838C >T locus of the DGAT1 gene, and the mutation comes from his father. Singer verification in case 2: (**C**) shows that the child has a mutation at the c.1215_1216delAG site of DGAT1 gene after singer verification, and the mutation comes from his father. The child shown in (**D**) had a mutation in the c.1049C >T locus of the DGAT1 gene, and the mutation originated from his mother.

The child presented with severe intractable diarrhea, malnutrition, and growth retardation. Therefore, we considered the possibility of congenital diarrhea. After obtaining informed consent from the baby's infant's family, 2 ml of peripheral blood from the child and her parents was collected for gene detection using a high-throughput sequencer (Beijing Zhiyin Oriental Gene Technology Co., Ltd.). There were two heterozygous mutations in the DGAT1 gene of type 7 congenital diarrhea, c.1215_c.1216delAG (p. Phe408fsTer74), c.838C >T (p. Arg280Ter, 209). To date, two concurrent mutations have not been reported. Singer sequencing confirmed that the mutation sites were from the father and mother, and were identified as compound heterozygous mutations (Figure 1). The c.1215_c.1216del AG mutation resulted in a frameshift mutation in exon 15 of DGAT1, encoding a protein with a stop codon at the 74th amino acid starting from phenylalanine at position 408. c. 838C >T site mutation induces a nonsense mutation in exon 9 of DGAT1. The corresponding protein had a mutation in arginine at position 280, which translated to a stop codon, making it impossible to translate the remaining 209 amino acids, shortening the protein by 209 amino acids.

**Figure 2:**
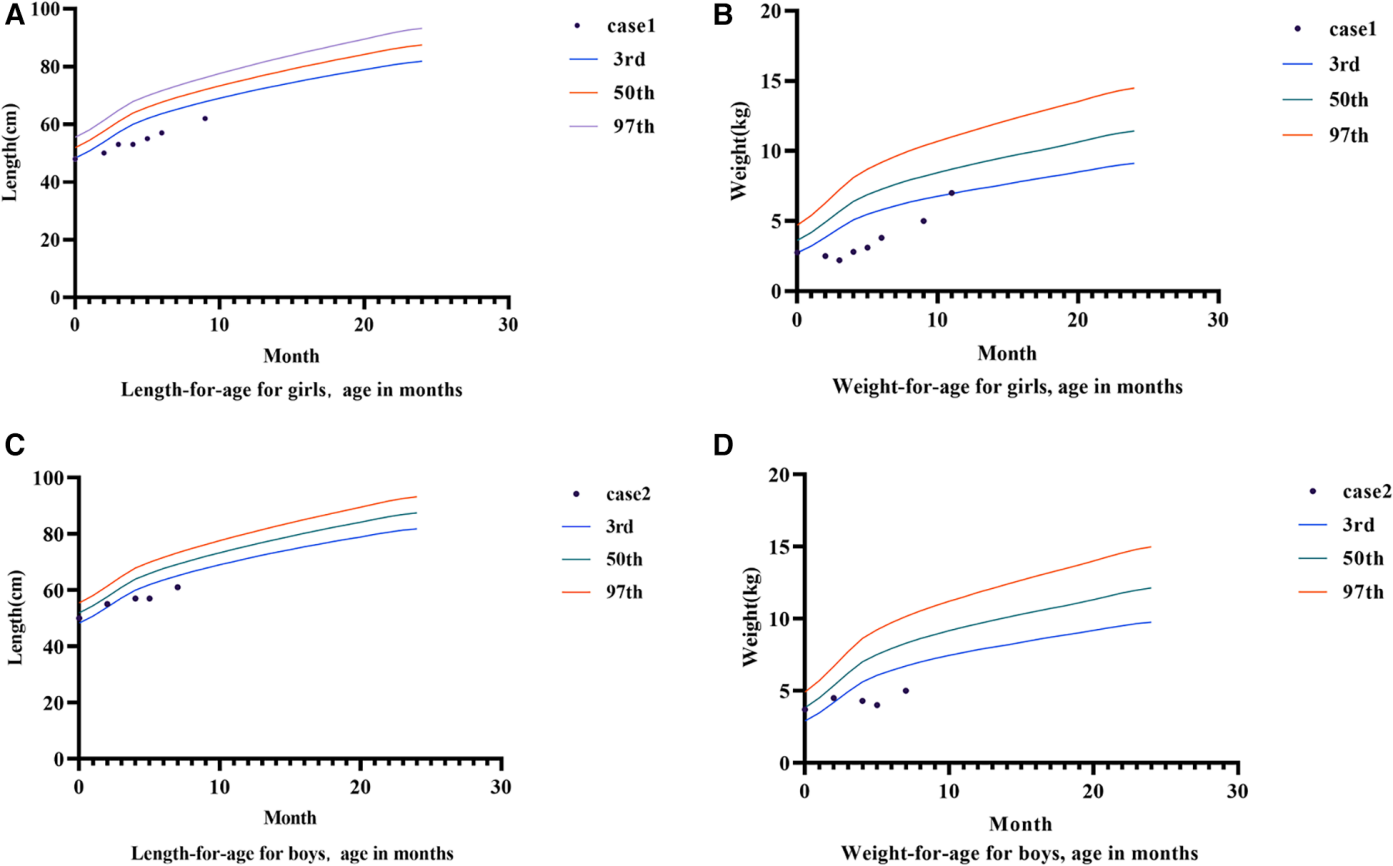
Growth charts of case 1 and case 2. (**A**) Shows the growth curve of case 1. (**B**) Is the weight growth curve of case 1. (**C**) Shows the growth curve of case 2. (**D**) Shows the weight growth curve of case 2.

Follow-up: After the gene detection results were revealed, the diagnosis was changed to congenital diarrhea (DGAT1 gene defect). The family was instructed to provide a whole-protein formula diet with 87% MCT (40% of the total energy provided by fat). Low-fat diets such as minced meat and rice flour were gradually supplemented when she was 5 months old. Diarrhea had not occurred since, and the infant manifested improved nutrition, with her weight increasing to 3.1 kg at 5 months. The patient's motor development was normal. At six months, complementary food, mainly a low-fat diet, was added to the infant's diet. However, the body weight growth was slow; therefore, her diet was transitioned to a low-fat (3% of total energy provided by fat) amino acid formula powder, and her body weight increased to the 3rd percentile at 11 months (Figure 2).

### 3.2. Case 2

**Figure 3:**
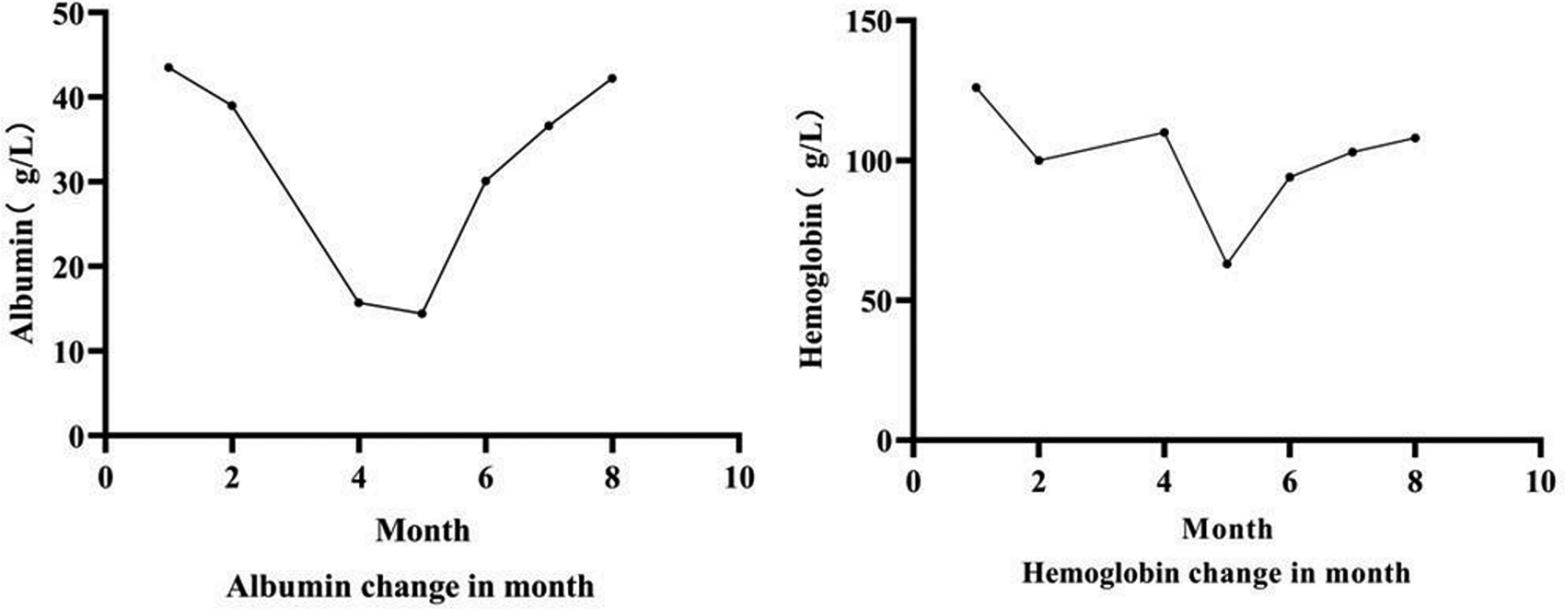
Trend chart of hemoglobin and albumin in case 2.

Diagnosis and treatment: The patient presented with severe intractable diarrhea and hypoproteinemia. Therefore, we suspected protein-losing enteropathy. Genetic testing was performed after informed consent was obtained from the patient's family. The gene detection results revealed that DGAT1 carried two heterozygous mutations, c.1215_c.1216delAG (p. Phe408fsTer74) and c.1049C >T (p. Ala350Val). The c.1049C >T mutation had not yet been identified. Singer sequencing confirmed that the mutations originated from the father and mother, demonstrating compound heterozygous mutations (Figure 1). The c.1215_c.1216del AG mutation resulted in a frameshift mutation in exon 15 of DGAT1, encoding a protein with a stop codon at the 74th amino acid starting from phenylalanine at position 408. c. The 1049C >T site mutation causes a mistranslation in exon 13 of the *DGAT1* gene, encoding a protein with alanine mutated to valine at position 350. The patient was diagnosed with congenital diarrhea (*DGAT1* gene defect), severe protein-energy malnutrition, hypoalbuminemia, hyponatremia, and hypokalemia. After being fed a whole protein formula powder with 87% MCT, the patient developed bloody stools The patient was then switched to an extensively hydrolyzed formula with 50% MCT, and the hematochezia was relieved. However, the infant still had diarrhea, vomiting, poor weight gain, and oedema in both lower limbs. Extensively hydrolyzed protein high-MCT formula powder (50% MCT content) was continuously pumped for 24 h. Albumin, immunoglobulin, and filtered white and red blood cell suspensions were infused, and sodium, potassium, and calcium supplement were administered to correct the positive-ion disorder. As a result, the frequency of diarrhea diminished, while oedema symptoms in both feet did not improve. Hypoalbuminemia and ion. disturbances recurred. The patient's diet was then adjusted for enteral nutrition with a low-fat amino acid formula powder (3% of the total energy provided by fat). Two weeks later, the diarrhea was significantly relieved with yellow paste-like stool 1–2 times daily. Re-examination demonstrated normal albumin and serum ion levels, with no recurrence of anemia. The infant was followed up until 7 of age; no diarrhea was observed, and his body weight increased to 5 kg (Figures 2, 3).

## 4. Discussion

To conduct a review: of the existing literature, “DGAT1”, “diarrhea,”, and “hypoproteinemia” were used as keywords when searching Pubmed. We retrieved 12 articles containing 29 cases of congenital diarrhea (4–15) caused by DGAT1 defects, of which three cases were reported in China. A total of 25 mutation sites related to DGAT1 were detected (Table 3), including eight splice site mutations, six missense mutations, five frameshift mutations, four full-length mutations, one insertion/deletion mutation, and one nonsense mutation. Among the 29 cases, there were 20 homozygous mutations, 8 compound heterozygous mutations, and 1 single heterozygous mutation. All children had onset in the neonatal period or early infancy, including 18 cases (62%) in the neonatal period and 11 cases (38%) within the 1st-4th month after birth. The initial symptoms were diarrhea (*n* = 25, 86%), vomiting (*n* = 21, 72%), vomiting without diarrhea (*n* = 4, 14%), growth retardation (*n* = 22, 76%), hypoalbuminemia (*n* = 18, 62%), hypertriglyceridemia (*n* = 6, 21%), and edema (*n* = 5, 17%). Three deaths occurred and 23 were mitigated. Twenty-three patients received parenteral nutrition therapy during hospitalization; 18 patients switched to low-fat or fat-free diets after their diarrhea or vomiting improved and exhibited continuous amelioration. Three patients were transitioned to a high-MCT formula and tolerated it well. However, one patient did not respond well to the high-MCT diet, and the other two recovered to regular diets. Six children were not administered parenteral nutrition therapies, and their diarrhea was relieved by altering their diet, including three cases of a high-MCT diet, one case of amino acid an formula, and two cases of a hydrolyzed formula.

**Table 3:** Mutation identified within *DGAT1* for congenital diarrhea.

No.	Mutant site	Amount	Mutation type	Clinical manifestations	Treatment	Prognosis
1	c.751 + 2 T >C	5	Homozygous	Vomiting, diarrhea, malnutrition, hypertriglyceridemia, hypoproteinemia	TPN, gastrointestinal fistula, amino acid formula feeding, regular infusion of albumin	Die
				Diarrhea, hypertriglyceridemia, hypoproteinemia	TPN, amino acid formula feeding, regular infusion of albumin	Thrive at 46 months without dietary restrictions
				Diarrhea, growth retardation, edema, hypotonia	TPN, amino acid formula feeding, oral rehydration salts	Symptoms improved, regular diet, diet except dairy products
				Diarrhea, hypertriglyceridemia	TPN, oral rehydration salts	Enteral nutrition maintain body weight and albumin
				Diarrhea, vomiting, growth retardation, hypoproteinemia, vitamin D deficiency	TPN, regular infusion of immunoglobulin, vitamin D supplement	Low-fat diet (Tolerex formula powder), diarrhea symptoms improved
2	c.884T >C	1	Homozygous	Diarrhea, edema, growth retardation, hypertriglyceridemia, hypoproteinemia	Amino acid formula feeding, regular infusion of albumin and immunoglobulin	No diarrhea at discharge
3	c.314T >C	2	Homozygous	Identical twins, diarrhea, hypertriglyceridemia, and growth retardation.	PN, gastrostomy, hydrolysis formula feeding, regular infusion of red blood cells and immunoglobulin	Low-fat diet (fat provides no more than 10% energy), the symptoms improved
4	c.1202G > A	1	Homozygous	Diarrhea, vomiting, growth retardation, hypoproteinemia	A diet free of fat and medium-chain triglycerides, with regular infusion of albumin	Low-fat diet and medium-chain triglycerides and fat-free formula feeding, diarrhea improved, normal growth
5	c.573_574delAGinsCCCATCC CACCCTGCCCATCT	1	Homozygous	Diarrhea, vomiting, growth retardation, hypoproteinemia, hypertriglyceridemia	TPN, infusion albumin	Diarrhea improve based on formula feeding
6	c.937-1G >A	1	Homozygous	Diarrhea, vomiting, growth retardation, hypoproteinemia	Hydrolyzed formula feeding	Diarrhea improved
7	c.953insC	1	Homozygous	Diarrhea, vomiting, growth retardation, hypoproteinemia	Hydrolysis formula feeding, oral lipase	Diarrhea has improved but remains malnourished
8	c.629_631delCCT	4	Homozygous	vomit	Infusion of fat-soluble vitamins	Fat-free enteral feeding, symptoms improved
				Vomiting, growth retardation	Infusion of fat-soluble vitamins	Fat-free enteral feeding, symptoms improved
				Vomiting, diarrhea, growth retardation, hypoproteinemia	TPN, small bowel transplantation	Diarrhea improved, growth retardation
				Vomiting, diarrhea, growth retardation, hypoproteinemia	TPN, fat-free formulation	Diarrhea improved, growth retardation
9	c.1013_1015delTCT C.1260C >G	1	Compound heterozygous	Vomiting, diarrhea, growth retardation, hypoproteinemia	TPN, amino acid formula by nasojejunal tube feeding	Low-fat diet, symptom improvement
10	c.895-1G >A	1	Homozygous	Vomiting, diarrhea	TPN	Die
11	c.1249-6 T >G	1	Homozygous	Diarrhea, edema, hypoproteinemia	TPN, enteral nutrition, infusion of albumin and immunoglobulin	Die
12	c.629_631delCCT	1	Homozygous	Diarrhea, vomiting, growth retardation, edema, hypotonia	TPN, gastrostomy, infusion of albumin and immunoglobulin	Low-fat formula (100% amino, 2% fat), feeding tolerance, no growth restriction
13	c.1310A >G c.981 + 1G >T	1	Compound heterozygous	Vomiting, growth retardation, vitamin D deficiency, anemia, ion disorder	TPN. Supplementation with vitamin D through the intestine does not respond, but vitamin D supplementation through the skin does	Symptom improvement
14	c.676 + 1G >A	1	Homozygous	Diarrhea, vomiting, anemia, vitamin D deficiency, rectal bleeding	TPN, vitamin D supplementation	Combination of Vivonex formula powder and baby food with limited solid matter intake, diarrhea improved
15	c.1311 + 1G >A c.1462delG	1	Compound heterozygous	Vomiting, growth retardation, hypogammaglobulinemia	TPN, regular infusion immunoglobulin	Enteral feeding with Enfaport formulation (containing 87% medium-chain triglycerides) improved vomiting and growth retardation
16	c.676 + 1G >T c.367_368delCT	1	Compound heterozygous	Vomiting, diarrhea, growth retardation, anemia	TPN, deep hydrolysis formula by nasal feeding, infusion of albumin	medium-chain triglycerides diet and low-fat diet, no diarrhea
17	c.288 + 1del c.629_631del	1	Compound heterozygous	Diarrhea, vomiting, growth retardation, hypogammaglobulinemia, ion disorder	TPN, supplementary immunoglobulin	Poor uptake of medium-chain triglycerides, low fat modular diet, diarrhea and growth retardation are reduced after gastrostomy
18	c.428_429del c.629_631del	1	Compound heterozygous	Vomiting, diarrhea, growth retardation, hypoproteinemia	TPN	Hydrolysis formula intolerance, gastrostomy, low fat modular diet, avoid high fat food and red meat, don't like dairy products, diarrhea and growth retardation improved
19	c.629_631del	1	Heterozygous	Diarrhea, hypoproteinemia, and growth retardation at 6 weeks of age.	TPN, gastrostomy	The symptoms diminished after a fat-free diet and subsequently developed celiac disease and IBD-like inflammation
20	c.838C >T c.1162C >T	2	Compound heterozygous	Diarrhea, vomiting, growth retardation, hypoproteinemia	TPN, hydrolyzed formula feeding	Low-fat diet, diarrhea and nutritional status improved.
21	c.838C >T c.1215_1216delAG	1	Compound heterozygous	Diarrhea, growth retardation, hypogammaglobulinemia	TPN, enteral high medium-chain triglycerides formula feeding	No diarrhea and improved nutritional status after Vivonex formula and low-fat diet
22	c.1215_1216delAG c.1049C >T	1	Compound heterozygous	Diarrhea, vomiting, hypoproteinemia, edema, rash, ion disorder	There was no improvement in symptoms in the high-chain triglyceride formula of gastric tube feeding. Diarrhea is reduced after feeding with vivonex formula. infusion of albumin	Diarrhea alleviated after replacement of vivonex formula and low-fat diet

Congenital diarrhea is a rare cause of severe chronic diarrhea in children. It often occurs during the neonatal period or in early infancy as a class of single-gene hereditary diseases. With progress in genome sequencing, many genes causing congenital diarrhea have been identified, including DGAT1. DGAT1 is located on chromosome 8 and encodes the DGAT1 and DGAT2 enzymes. In the human small-intestine epithelium, DGAT1 is the only enzyme that is highly- expressed enzyme. DGAT1 is a key enzyme that converts diacylglycerol and fatty acyl-CoA into triacylglycerol during fat absorption in the small intestine. Triacylglycerol is a critical molecule in energy storage and lipid metabolism and is a major component of breast milk fat. DGAT1 deficiency is a rare autosomal recessive genetic disease first reported by Haas et al. in 2012 (4). It is characterized by early-onset watery diarrhea and protein-losing enteropathy and is classified as a type 7 congenital diarrhea disease. Its pathogenic mechanism remains unclear but which may be attributed to diet-derived DGAT1 lipid substrate accumulation in the intestine, causing lipotoxic stress in the intestinal cells and resulting in cell dysfunction (5, 16). It may also be related to the deletion of transporters and connexins in the apical region of intestinal epithelial cells and a reduction in various enzymes (e.g., SGLT1, CD10, DPPIV, and NHE3) (6).

Diarrhea and protein-losing enteropathy induced by DGAT1 gene mutations are infrequent and are mostly reported abroad. Two of the three cases reported in China were diagnosed as protein-losing enteropathy, and these two cases manifested gene defects through gene detection and died clinically. The third study reported a detailed diagnosis and treatment process. Given that chronic diarrhea caused by genetic mutations is clinically infrequent, patients are difficult to be diagnosed in the early stages and are often misdiagnosed with infections, allergies, etc. Case 2 in this study was also initially misdiagnosed with a cow milk protein allergy. Patients who exhibit no significant improvement after routine treatment should undergo gene detection for a definitive diagnosis.

After excluding patients with diarrhea due to common causes, the two patients in this study underwent gene detection in the early stage of the disease. The results revealed compound heterozygous mutations in DGAT1 derived from the parents. There were three mutations: a nonsense mutation c.838C >T, a frameshift mutation c.1215_1216delAG, and a missense mutation c.1049C >T. These three mutations were all *de novo*, reported for the first time, and enriched the existing DGAT1 variant database. Here, two patients were intervenated with targeted therapies after gene detection, which showed remarkable efficacy. Hence, early targeted treatment can significantly improve the disease prognosis.

However, there are deficiencies in this diagnostic process. Neither case was subjected to enteroscopy or pathological examination; thus, DGA1 protein expression in intestinal epithelial cells could not be assessed, considering that (7) diarrhea symptoms in children are correlatedwith the amount of residual DGAT1 enzyme activity in intestinal epithelial cells. One study emphasized the importance of immunohistochemistry in the diagnosis of rare diseases (8). It describes an infant developing diarrhea, growth retardation, and hypoalbuminemia 6 weeks after birth. Gene detection revealed a single heterozygous mutation in DGAT1 (c.629_631delCCT), according to which type 7 congenital diarrhea could not be diagnosed. Immunohistochemistry confirmed the complete loss of DGAT1 protein expression in the intestinal epithelial cells and hepatocytes, consistent with DGAT1 gene inactivation.

Currently, no therapeutic agents are available to increase or restore intestinal DGAT activity in children with a DGAT1 deficiency. Most dietary interventions are based on the experience of a few confirmed patients (9): Low-fat or fat-free diets are primajorily employed to alleviate clinical symptoms, but their efficacy may be inconsistent due to varying mutation sites and the degree of protein synthesis disorder. In the acute phase, symptomatic treatments, such as parenteral nutrition, albumin and gamma-globulin supplementation, and/or red blood cell transfusion, were administered. In the literature, despite a shared mutated gene, 29 cases manifested various phenotypic penetrances, and their symptoms were associated with the amount of residual DGAT1 enzyme activity (7). Patients with a complete loss of DGAT1 enzyme activity can tolerate only 4%–7% of the total energy provided by fat, whereas patients with partial residual DGAT1 enzyme activity can tolerate 10% (9). In this study, the diet in case 1 was supplemented with a high-MCT hydrolyzed formula (40% of the total energy provided by fat). Although the patient did not exhibit feeding intolerance or diarrhea, her body weight did not increase. The low-fat formula resulted in a favorable weight gain. In case 2, the high-MCT extensively hydrolyzed formula (40% of the total energy provided by fat) did not mitigate diarrhea, whereas the low-fat formula improved diarrhea with good weight gain. Therefore, diets in the early stages of the disease should be predominantly low-fat formulas and then transitioned to high-MCT feeding after symptom relief and weight gain to obtain a good prognosis. In addition to low-fat or fat-free diets, a high MCT diet is suitable for infants with DGAT1deficiency. The reason for this is elusive and may be related to residual DGAT1 activity in intestinal epithelial cells or the direct absorption of MCT into the portal vein (17) by intestinal cells.

DGAT1-deficient infants often have an onset in the neonatal period and early infancy, and present with severe symptoms. The disease can be life-threatening if not treated appropriately; therefore, early diagnosis is crucial. For infants with unexplained diarrhea and vomiting accompanied by growth retardation and protein-losing enteropathy, DGAT1 deficiency should be highly suspected. Early genetic detection and pathological intestinal examinations can be used for an early diagnosis.

## 5. Patient perspective

In the manuscript, the parents have given their provided written informed consent to publish their cases.

## Data Availability

The datasets presented in this study can be found in online repositories. The names of the repository/repositories and accession number(s) can be found in the article/Supplementary Material.
